# Mig1 localization exhibits biphasic behavior which is controlled by both metabolic and regulatory roles of the sugar kinases

**DOI:** 10.1007/s00438-020-01715-4

**Published:** 2020-09-19

**Authors:** Gregor W. Schmidt, Niek Welkenhuysen, Tian Ye, Marija Cvijovic, Stefan Hohmann

**Affiliations:** 1 Department of Biosystems Science and Engineering, ETH Zurich, Basel, Switzerland; 2 Department of Chemistry and Molecular Biology, University of Gothenburg, Göteborg, Sweden; 3 Department of Mathematical Sciences, University of Gothenburg and Chalmers University of Technology, Göteborg, Sweden; 4 Department of Biology and Biological Engineering, Chalmers University of Technology, Göteborg, Sweden

**Keywords:** Microfluidic, Yeast, Glucose repression, Hexokinase, Hexose, Localization, Oscillation, Mig1

## Abstract

Glucose, fructose and mannose are the preferred carbon/energy sources for the yeast *Saccharomyces cerevisiae*. Absence of preferred energy sources activates glucose derepression, which is regulated by the kinase Snf1. Snf1 phosphorylates the transcriptional repressor Mig1, which results in its exit from the nucleus and subsequent derepression of genes. In contrast, Snf1 is inactive when preferred carbon sources are available, which leads to dephosphorylation of Mig1 and its translocation to the nucleus where Mig1 acts as a transcription repressor. Here we revisit the role of the three hexose kinases, Hxk1, Hxk2 and Glk1, in glucose de/repression. We demonstrate that all three sugar kinases initially affect Mig1 nuclear localization upon addition of glucose, fructose and mannose. This initial import of Mig1 into the nucleus was temporary; for continuous nucleocytoplasmic shuttling of Mig1, Hxk2 is required in the presence of glucose and mannose and in the presence of fructose Hxk2 or Hxk1 is required. Our data suggest that Mig1 import following exposure to preferred energy sources is controlled via two different pathways, where (1) the initial import is regulated by signals derived from metabolism and (2) continuous shuttling is regulated by the Hxk2 and Hxk1 proteins. Mig1 nucleocytoplasmic shuttling appears to be important for the maintenance of the repressed state in which Hxk1/2 seems to play an essential role.

**Electronic supplementary material:**

The online version of this article (10.1007/s00438-020-01715-4) contains supplementary material, which is available to authorized users.

## Introduction

**Fig. 1:**
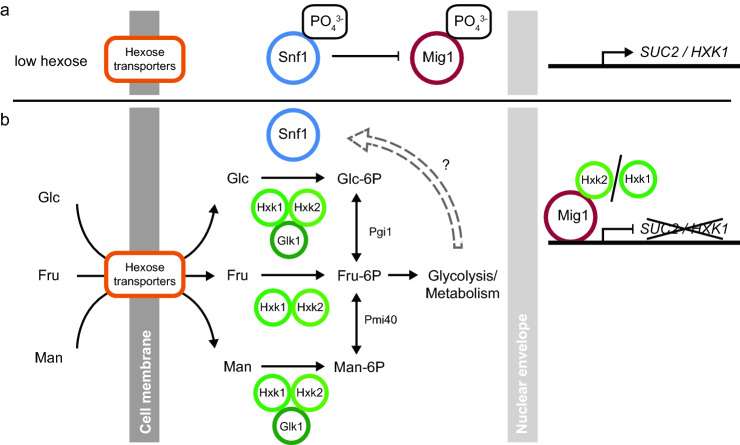
The carbon catabolite repression pathway. **a** When hexoses are absent, the Snf1 kinase is active and phosphorylates Mig1, which localizes to the cytoplasm. This enables expression from promoters, such as the *SUC2* and *HXK1* promoter. **b** Available hexoses are transported into the cell by hexose transporters and subsequently phosphorylated by Hxk1, Hxk2 and/or Glk1 enzymes. All hexose-phosphates are isomerized to fructose-6-phosphate and enter glycolysis. Snf1 kinase is inhibited through unknown mechanisms, which relieves the inhibitory phosphorylation of Mig1 and allows it to act as a transcriptional repressor of *SUC2* and *HXK1* expression in the nucleus. Hxk2 takes part in repression of *SUC2* in the nucleus

The ability to sense and appropriately respond to the availability of nutrients is a central feature of all living organisms. The yeast *Saccharomyces cerevisiae* has evolved a complex signal transduction network for the sensing of carbon/energy sources and maintenance of energy homeostasis. Availability of the preferred energy source glucose (or fructose, mannose) mediates, among others, catabolite repression of genes whose products are required for utilization of alternative energy sources via the transcriptional repressor Mig1 (Gancedo 1992; Thevelein 1994). When the preferred energy sources become limiting the Snf1 kinase is phosphorylated and activated (Schmidt and McCartney 2000; Mayer et al. 2011; Xiao et al. 2011; Chandrashekarappa et al. 2013). Activated Snf1 phosphorylates Mig1 which is exported from the nucleus to the cytosol (Treitel et al. 1998; DeVit and Johnston 1999), leading to derepression of genes for utilization of alternative energy sources (Fig. 1a). When preferred energy sources become available, Snf1 and Mig1 are dephosphorylated (Ludin et al. 1998; Ruiz et al. 2011, 2013) and Mig1 re-enters the nucleus and represses transcription of target genes by recruiting the repressor complex Tup1/Ssn6 (Treitel and Carlson 1995) (Fig. 1b). Time-lapse imaging has revealed that under constant growth conditions a large fraction of Mig1 moves in and out of the nucleus in a pulsatile fashion (Dalal et al. 2014; Lin et al. 2015) and that Mig1’s nucleocytoplasmic localization may be governed through the interaction with other proteins (Wollman et al. 2017).

The metabolic enzyme hexokinase 2 (Hxk2) has long been known to be required for glucose repression (Entian and Fröhlich 1984) and it is required for nuclear localization of Mig1 in the presence of glucose (Moreno and Herrero 2002; Ahuatzi et al. 2007). *S. cerevisiae* encodes three hexokinases that can produce hexose-6-phosphates: Hxk1, Hxk2, which phosphorylate glucose, fructose and mannose, and Glk1, which is glucose- and mannose-specific. Hxk2 is the main glucose phosphorylating enzyme when glucose is abundant (Herrero et al. 1995). Hxk2 has been reported to accumulate in the nucleus where it may interact with Mig1, but the physiological relevance of these observations remains unclear (Randez-Gil et al. 1998; Ahuatzi et al. 2004). Overexpression of Hxk1 in an *hxk2∆* background partly restores glucose repression while any one of the hexokinases is sufficient for fructose repression (Kraakman et al. 1999). This indicates that the Mig1-mediated catabolite repression depends on the specific hexose and the hexose phosphorylating enzymes. The fact that both hexokinases (I) act as metabolic enzymes, (II) are potentially involved in gene regulation (Rose et al. 1991; Vega et al. 2016), (III) are differentially expressed (Sierkstra et al. 1992; Herrero et al. 1995) and (IV) appear to regulate each other’s expression (De Winde et al. 1996; Rodriguez et al. 2001) has complicated the elucidation of their respective role in catabolite repression.

When one of the preferred hexoses becomes available catabolite repression proceeds through two distinct phases (De Winde et al. 1996; Sanz et al. 1996). The first phase requires the presence of hexose phosphorylation activity by any sugar kinase, including Glk1 (De Winde et al. 1996; Sanz et al. 1996). In adapted cells, Hxk2 is required to maintain glucose repression (Entian and Fröhlich 1984; De Winde et al. 1996; Sanz et al. 1996) and either Hxk1 or 2 are required in fructose. Although Mig1 is dephosphorylated during the first phase of the catabolite repression response, it remains dephosphorylated during the second phase only under high glycolytic rates (Elbing et al. 2004). Most studies on catabolite repression employed gene expression or enzyme activities as read-outs. More recent studies have focused on understanding the dynamics of catabolite repression using fluorescence imaging of Mig1 localization in living cells. It appears that the short and long-term response of Mig1 localization may be governed by distinct mechanisms.

Here, we study the dynamics of Mig1 nucleocytoplasmic localization in different hexokinase mutants during a switch from de-repressed to different catabolite repressing conditions. We link Mig1 patterns to catabolite repression by studying fluorescent reporter gene constructs of the catabolite repressed promoters *SUC2* and *HXK1* under the same conditions. We demonstrate that Mig1 nuclear localization and catabolite repression exhibit two distinct phases after a switch to repressing conditions. In the first phase, a large fraction of cellular Mig1 localizes to the nucleus, an effect facilitated by any of the sugar kinases. In the second phase, pulsatile nucleocytoplasmic shuttling of Mig1 is observed, which requires Hxk2 in glucose and mannose, whereas either Hxk1 or Hxk2 can confer nucleocytoplasmic shuttling in fructose. Hence it appears that carbon catabolite repression depends on (I) the ability of Mig1 to localize to the nucleus and (II) the presence of the correct hexokinase(s) depending on the hexose in the environment.

## Materials and methods

### Strains and plasmids

The yeast strains were transformed with GFP-KanMX and mCherry-hphNT1 using standard methods for yeast genetics and transformation (Gietz and Woods 2002). Yeast strains were grown to mid-log phase at 30 *°*C in YNB synthetic complete medium containing 1.7 g/l yeast nitrogen base, 5 g/l ammonium sulfate, 670 mg/l complete supplement mix; 10 mg/l adenine and supplied with 540 mM ethanol overnight. An overview of the strains and plasmids used is available in the electronic supplementary material.

### Setup of microfluidic imaging experiments

The cells were inoculated in 5 mL of CSM media (lacking the appropriate amino acids for auxotrophic selection) supplemented with 3% ethanol and 0.05% glucose from freshly streaked (2–4 days old) plates and incubated in a shaker at 270 rpm and 30 *°*C for at least 8 h on the day before the experiment. In the evening cells were diluted to OD_600_ 0.5 or 1.0 in fresh media for overnight incubation. On the next morning, the cell concentration was measured using a Z2 Coulter Counter (Beckman Coulter, Nyon, Switzerland). Typical cell concentrations ranged from 5 to 15 * 10^6^ cells/ml. Cells were centrifuged and the supernatant was removed to yield a final cell concentration of 20 * 10^6^ cells/ml and cells where loaded on the microfluidics chip. The microfluidic chip used was adapted from Frey et al. (2015). Cell imaging in the microfluidics chips was performed on a Nikon Ti Eclipse (Nikon Instruments) inverted fluorescence microscope, placed in an environmental box (Life Imaging Services, Switzerland) to maintain the temperature at 30 *°*C. We used a 40× Plan Fluor Oil DIC N2 objective (MRH01401, Nikon AG, Egg, Switzerland). The microscope was equipped with an ORCA Flash 4.0 camera (Hamamatsu Photonic, Solothurn, Switzerland). The microscope was equipped with a Spectra X Light Engine fluorescence excitation light source (Lumencor, Beaverton, USA) and a pE-100 brightfield light source (CoolLED Ltd., UK). Hardware triggering between the light sources and the camera was implemented using an Arduino UNO (Somerville, MA, USA), to avoid any additional illumination of the sample due to hardware delays. All measurements were run with a diffuser and a green interference filter placed in the brightfield light path. The perfect focus system of the microscope was enabled for all measurements. The microscope was controlled using YouScope (Lang et al. 2012). Images were acquired at different intervals throughout the time course. In the time before the media switch (− 240 to 0 min) images were acquired every 30 min. After the media change images were acquired at an interval of 5 min (0 to 720 min). Brightfield images were acquired above and below the focal plane (Nikon Perfect Focus System, ± 5 AU) to facilitate cell segmentation. Fluorescence images of the fluorescent protein mAmetrine, which is excited using blue light, were acquired at only every fourth time-point of the experiment to prevent photodamaging the cells. All other fluorescent proteins were imaged at every time-point. The filter cubes, exposure time and light intensities for all imaging channels used can be found in Suppl. Table 4. Optical filters were purchased from AHF Analysentechnik AG (Tübingen, Germany). Additional information on the fabrication and operation of this microfluidic chip, sample preparation and imaging is provided in the electronic supplementary material.

### Image analysis

Brightfield images acquired above the focal plane were divided by images acquired below the focal plane using custom Matlab scripts. Division of images leads to elimination of uneven illumination and enhances diffraction pattern of cells. Segmentation was performed on the resulting images using CellX (Mayer et al. 2013). The Mig1-localization index was calculated from the CellX output as follows:

$${\text{Localization}}_{\text{index}} = \frac{{{\text{Medianf}}_{\text{nuc}} }}{{{\text{Medianf}}_{\text{total}} }} - 1.$$

Cells were tracked using custom Matlab scripts as described previously (Ricicova et al. 2013).

### Invertase assay

Cells were grown in YNB 4% glucose overnight and shifted to the appropriate carbon-source concentration. Samples were harvested by centrifugation and washed three times with ice-cold water. Protein extracts and measurements of invertase activity were performed as described previously (Hohmann and Zimmermann 1986). The protein concentration was determined by using the DC protein assay kit (Bio-Rad, Hercules, CA).

### Glucokinase assay

Cells were grown on YNB 4% glucose overnight and samples were harvested by centrifugation and washed three times with ice-cold water. Proteins were extracted and the glucokinase activity was measured as described previously (Lobo and Maitra 1977). The protein concentration was determined by using the DC protein assay kit (Bio-Rad, Hercules, CA).

## Results

### All hexose kinases contribute to Mig1 nuclear localization

**Fig. 2:**
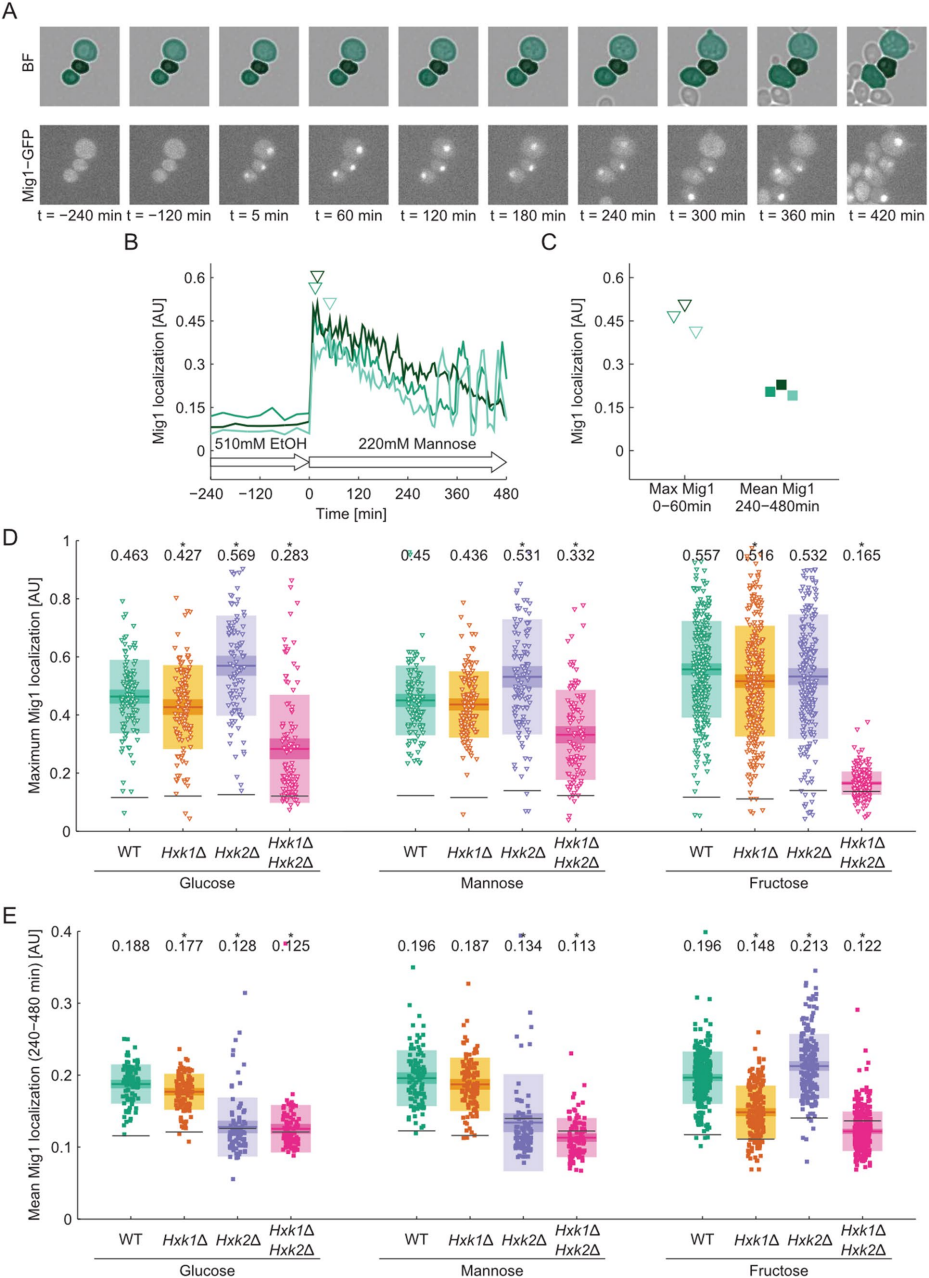
**a** Time-lapse images of Mig1-GFP tagged wild type cells in brightfield (overlaid with segmentation mask) and GFP channel. Cells were shifted from ethanol (510 mM) to mannose (220 mM) at 0 min. **b** Quantification of Mig1 localization from data in a. The Mig1 localization index was calculated as the fraction of cellular Mig1 present in the nucleus (see image analysis section of materials and methods for details). The time-point of maximum Mig1 localization from 0 to 60 min after the media shift for each cell is marked with open triangles. Mig1 localization pulse maxima are indicated by closed triangles. **c** Metrics extracted from single cell traces in **b**. The maximum and mean Mig1 localization from 0 to 60 min after the media shift are shown with open symbols. The mean Mig1 localization pulse height for each cell and the mean overall localization of Mig1 from 240 to 480 min after the media shift are shown with closed symbols. **d** Maximum Mig1 localization from 0 to 60 min and **e** time-averaged Mig1 localization from 240 to 480 min after shift from ethanol to 220 mM of the indicated hexose in wild type and hexokinase mutants. Each point represents a single cell measurement. Colored horizontal lines indicate the mean, and the 95% confidence interval and one standard deviation are shown in consecutively lighter hues of the respective color. The mean value is given in the top of the graph and significance is indicated by star (*p* < 0.05, paired *T* test against wild type condition). Black horizontal lines indicate the basal Mig1 localization level as determined from the time points before the media shift

We measured in living cells over time the nucleocytoplasmic localization of Mig1-GFP as a read-out for the dynamics of the Snf1–Mig1 system (De Vit et al. 1997). Large values indicate Mig1-GFP nuclear localization, whereas low values denote cytosolic localization. When wild type cells pre-incubated with ethanol as a sole energy source were exposed to any of the three hexoses, Mig1 localized to the nucleus and reached its maximum nuclear localization signal within ~ 30 min (Fig. 2a). Subsequently, the Mig1 nuclear localization declined over 60–240 min, with a large cell-to-cell variability. Following this initial response, nucleocytoplasmic shuttling of Mig1 was observed as described earlier (Dalal et al. 2014; Lin et al. 2015) (Fig. 2b and Suppl. Movie 1). After switching the medium back to non-repressing conditions (510 mM ethanol, 480 min) Mig1 localized to the cytoplasm immediately as expected (Bendrioua et al. 2014) (Suppl. Figure 1). To compare the localization of Mig1 between strains and conditions, we quantified the maximum Mig1 localization in the hour directly after the shift (0–60 min, Fig. 2c) and the time-averaged Mig1 localization in adapted cells (240–480 min, Fig. 2c) for each single cell trace.

We monitored Mig1-GFP dynamics following shifts to the three hexoses in cells lacking Hxk1, Hxk2 or both. In all strains, Mig1 localized from the cytoplasm to the nucleus immediately after the cells were exposed to any of the hexoses, except for the *hxk1*∆*hxk2*∆ double mutant exposed to fructose (Fig. 2d). This strain/hexose combination is the only one where the sugar cannot be metabolized at all. At the same time, it appears that Glk1 is sufficient to trigger Mig1 nuclear localization after exposure to glucose and mannose (Lobo and Maitra 1977; Ma et al. 1989). The strength of the Mig1 response seems to be related to the specific activity of hexose phosphorylation. For instance, the *hxk1*∆*hxk2*∆ strain showed a short Mig1 nuclear localization of reduced amplitude in glucose and mannose, in line with the ability of Glk1 to phosphorylate these hexoses with a lower specific activity than the hexokinases (Lobo and Maitra 1977) (Suppl. Fig. S1). Deletion of *HXK1* did not affect the initial Mig1 localization following glucose addition, in line with the notion that expression of *HXK1* is repressed in the presence of Hxk2 (Rodriguez et al. 2001). Also, in the *hxk2*∆ strain the initial Mig1 localization maximum increased (Fig. 2d). Similar effects were observed when cells were shifted to mannose. After the shift to fructose, the initial Mig1 localization was largely unaffected by single hexokinase deletions (Suppl. Fig. S1).

Under all conditions tested Mig1 never stayed continuously localized to the nucleus, but oscillated between cytosol and nucleus. In contrast to the short-term behavior of Mig1, nuclear localization of Mig1 after adaption to repressing conditions (240–480 min) could only be observed in the presence of specific hexokinases but not in the *hxk1*∆*hxk2*∆ double mutant. In glucose, deletion of *HXK2* resulted in complete loss of Mig1 nuclear localization and nucleocytoplasmic shuttling in adapted cells (Fig. 2e and Suppl. Fig. S1). Similarly, Mig1 localization was abolished in *hxk2*∆ and *hxk1*∆*hxk2*∆ strains in mannose.

Taken together, it appears that the Mig1-GFP localization response can be divided into at least two phases based on the temporal behavior, the dynamics and the requirement for sugar phosphorylating enzymes. We observed a rapid initial nuclear accumulation of Mig1-GFP that requires hexose phosphorylation activity but no specific sugar kinase. In adapted cells, after the initial 60 min, Mig1-GFP remains predominantly nuclear, but oscillates between cytosol and nucleus and the shuttling intensity depends on the sugar and the hexose kinases present. Nuclear presence of Mig1-GFP in such adapted cells seems to require Hxk2 in the presence of glucose and mannose and any of the two Hxk1 or Hxk2 in the presence of fructose.

### Ectopic expression of any sugar kinase restores initial Mig1 localization, but only Hxk1 and Hxk2 restore sustained nuclear accumulation and nucleocytoplasmic shuttling

In glucose-repressed cells, expression of the sugar kinases is subject to feedback regulation by the hexokinases themselves (Rodriguez et al. 2001), which complicates the interpretation of results from experiments with single hexokinase deletions. To dissect the role of the Hxk1 and Hxk2 proteins, independent of their endogenous expression level and feedback regulation, we employed a *hxk1*∆*hxk2*∆ strain carrying endogenously tagged Mig1-GFP and a set of plasmids that express either *HXK1*, *HXK2* or *GLK1* under the control of a *TDH3*-promoter and *CYC1*-terminator. To quantify expression of each sugar kinase in single cells, the plasmids carried a second expression cassette with the fluorescent protein mAmetrine, also under the control of a *TDH3*-promoter and *CYC1*-terminator. Restoration of glucose phosphorylating activity to levels higher than in the wild type indicated that the kinases were functionally expressed and at a higher level than in the wild type (Suppl. Fig. S2). Again, we monitored Mig1 localization during a media shift from 510 mM ethanol to 220 mM of either glucose, fructose or mannose.

**Fig. 3:**
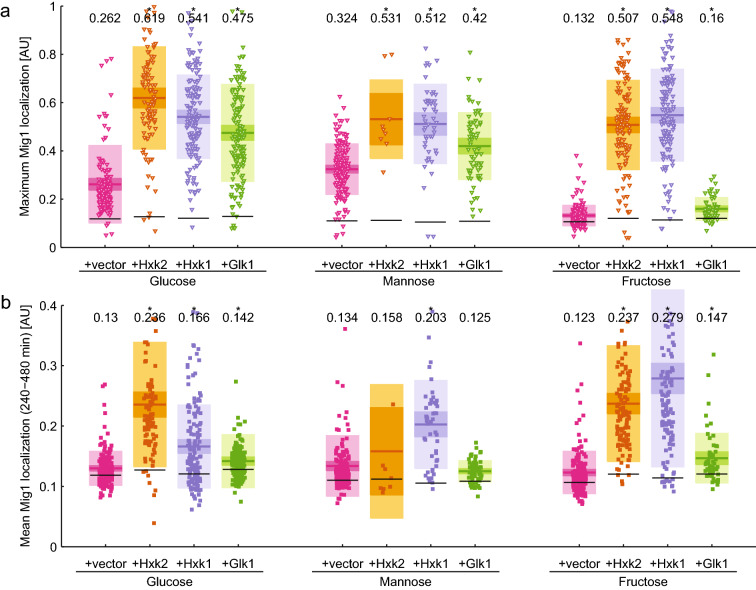
Short (**a**) and long-term (**b**) response of Mig1 localization after shift from ethanol to 220 mM of the indicated hexose in a *hxk1∆hxk2∆* background overexpressing one of the sugar kinases. Each point represents a single cell measurement. Horizontal lines indicate the mean, and the 95% confidence interval and one standard deviation are shown in consecutively lighter hues of the respective color. The mean value is given in the top of the graph and significance is indicated by a star (*p* < 0.05, paired *T* test against wild type condition). Black horizontal lines indicate the basal Mig1 localization level as determined from the time points before the media shift

The initial Mig1-GFP nuclear accumulation response after the media shift was restored by expression of any sugar kinase (Fig. 3a and Suppl. Fig. S3) to the same or even higher extent than in the wild type (Fig. 2d) except for Glk1 in the case of fructose. This was expected since this strain is unable to phosphorylate fructose.

Overexpression of the hexokinases resulted in the partial inability of the cells to divide or even cell death after the shift to mannose (Suppl. Movie 2 and 3). This effect was more pronounced for Hxk2 than for Hxk1 overexpressing cells (Suppl. Fig S4) and appeared when a threshold expression level of the sugar kinase was exceeded (Suppl. Fig. S5b). In mannose, the only viable cells showed low expression from the *TDH3*-promoter (mAmetrine signal < 1000 AU for +Hxk2 and < 1500 AU for +Hxk1) indicating that these cells had a low expression level of the hexokinases. In contrast, cells harboring the empty vector or plasmids overexpressing Glk were viable irrespective of the *TDH3*-promoter activity. We did not observe growth inhibition after the shift to glucose or fructose, even in cells that showed high mAmetrine levels. Hence it appears that in mannose strong overexpression of Hxk1 or Hxk2 is toxic to cells, probably because the control restricting sugar kinase activity is defective, similar to the situation in a *tps1Δ* mutant (Blázquez et al. 1993; Bonini et al. 2003).

After adaption to repressing conditions, nuclear accumulation and nucleocytoplasmic shuttling of Mig1 was restored by expression of Hxk1 or Hxk2, whereas Glk1 was unable to restore shuttling of Mig1 (Fig. 3b and Suppl. Fig. S3). We quantified the fluorescence signal of the *TDH3*-mAmetrine reporter as a measure for the sugar kinase expression level, presuming that the expression levels are correlated as both proteins are expressed from the same plasmid under the control of the same promoters and terminators. This measure of the expression level was plotted against the average Mig1 signal at 240–480 min after the media shift. For all hexoses, shuttling of Mig1 was restored in a dose-responsive manner correlating with the expression level of Hxk1 and Hxk2 (Suppl. Fig S5). In mannose, much lower expression levels of the hexokinases led to high Mig1 nuclear localization as compared to glucose and fructose.

In summary, the initial Mig1 nuclear accumulation in the *hxk1*∆*hxk2*∆ mutant upon energy source shifts could be restored by expression of any sugar kinase except for Glk1 in the case of fructose. Restoration of nucleocytoplasmic shuttling of Mig1 in the long-term response could only be accomplished by expression of one of the hexokinases. This suggests that the initial import of Mig1 depends on the presence of any hexose phosphorylating activity, whereas the subsequent nucleocytoplasmic shuttling requires the presence of Hxk1 or Hxk2 specifically.

### Nuclear localization of Mig1 is not correlated with catabolite repression

**Fig. 4:**
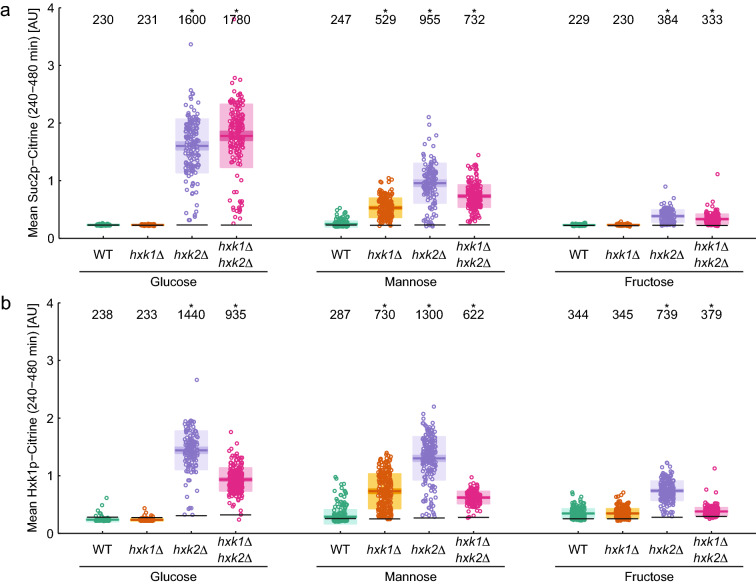
Expression from the *SUC2* (**a**) or *HXK1* (**b**) promoter after shift from ethanol to 220 mM of the indicated hexose in wild type (WT) and hexokinase mutants. Each point represents a single cell measurement. Horizontal lines indicate the mean, and the 95% confidence interval and one standard deviation are shown in consecutively lighter hues of the respective color. The mean value is given in the top of the graph and significance is indicated by a star (*p* < 0.05, paired *T* test against wild type condition). Black horizontal lines indicate the basal fluorescence level as determined from the time points before the media shift

To correlate Mig1-GFP nuclear dynamics with the expression status of Mig1 target genes we constructed a set of strains expressing a destabilized fluorescent protein under the control of the *SUC2* or *HXK1* promoter (see electronic supplementary material for details), respectively (Lutfiyya and Johnston 1996; Lutfiyya et al. 1998). We tested the functionality of these constructs by comparing the fluorescence signal to specific invertase activity measurements (Suppl. Fig. S6). We monitored the fluorescent signal by time-lapse imaging in the microfluidic device (Fig. 4).

Before the switch to glucose-containing media, during growth on ethanol, we did not detect expression from the *SUC2* and *HXK1* promoters, as these promoters are known to be repressed through a Mig1-independent mechanism involving Rgt1 in the absence of glucose (Weinhandl et al. 2014). After the switch to glucose-containing media, expression from the *SUC2* and *HXK1* promoters was observed in the *hxk2*∆ and *hxk1*∆*hxk2*∆ strains, whereas the wild type and *hxk1*∆ strains showed no activity from the *SUC2* and *HXK1* promoters (Suppl. Fig. S7 and S8), presumably because these promoters are partially repressed in the wild type and *hxk1*∆.

The earliest activation of the *SUC2* promoter could be observed ~ 30 min after the media shift in the *hxk1*∆*hxk2*∆ strain (Suppl. Fig. S7j and S8j), which is in the range of the maturation time of the expressed fluorescent protein Citrine. In the *hxk2*∆ strain, expression from both promoters was delayed to ~ 120 min after the media shift (Suppl. Fig. S7g and S8g). This indicates that glucose repression was functional during the short-term response even in the absence of Hxk2. Interestingly, the time points at which expression from the *SUC2* promoter was first detected coincided with the end of the initial Mig1 responses if one accounts for the additional lag-time due to maturation of Citrine (Suppl. Fig. S9). Also, in mannose and fructose, whenever expression from the *SUC2* and *HXK1* promoters could be observed, it was limited to the time after the initial localization response of Mig1 was completed (Suppl. Fig. S9).

To quantitatively compare the extent to which repression was established in the long-term response we quantified the time-averaged fluorescent signal for each single cell trace between 240 and 480 min after the shift to hexose-containing medium (Fig. 4). Complete repression of *SUC2* was observed in glucose for the wild type and *hxk1*∆ strains, whereas the *hxk2*∆ and *hxk1*∆*hxk2*∆ mutants showed derepression of *SUC2* (7–8-fold, Fig. 4a) as expected. A similar behavior was observed after the shift to fructose, although the expression of *SUC2* was weaker than in glucose (1.5-fold). Hence it appears that nuclear shuttling of Mig1 is sufficient to maintain repression of *SUC2* during the long-term response to glucose and fructose, whereas the absence of shuttling leads to derepression (Suppl. Fig. S9). In contrast, the shift to mannose resulted in derepression of the *SUC2* promoter in all strains to varying degrees. *SUC2* expression was strongest in the *hxk2*∆ single mutant (fourfold increase), followed by the double mutant (threefold increase) and the *hxk1*∆ strain (twofold increase, Fig. 4a). Also in wild type cells, the sporadic activity of the *SUC2* promoter-reporter could be detected, concurrent with the notion that even high concentrations of mannose cannot mediate full catabolite repression (Suppl. Fig. S7) (Dynesen et al. 1998). In mannose, the mechanism for long-term repression of *SUC2* appears to be different than for glucose and fructose. In mannose nucleocytoplasmic shuttling of Mig1 was not sufficient to maintain *SUC*2 repression, even though the mean long-term Mig1 localization in mannose was comparable to glucose (Fig. 2e). In summary, full repression of *SUC2* requires Hxk2 during growth on glucose, while growth on fructose and mannose only show a partial release of *SUC2* repression in absence of Hxk2 (and Hxk1 for mannose). While Hxk2 and Hxk1 are required to reach maximum repression of *SUC2* in mannose.

Repression of the *HXK1* promoter appeared to be less tight than that of the *SUC2* promoter. Sporadic expression from the *HXK1* promoter was observed in single wild type cells in all hexoses (Fig. 4b and Suppl. Fig. S8). The *hxk1*∆ strain behaved exactly like the wild type in glucose and fructose whereas in mannose deletion of *HXK1* led to a 2.5-fold increase in expression from the *HXK1* promoter. Expression from the *HXK1* promoter was highest in *hxk2*∆ strains in all hexoses, whereas the *hxk1*∆*hxk2*∆ strain reached only 50% of that level (Fig. 4b) or was even reduced to wild type levels in fructose (which this strain cannot metabolize). These data indicate that *HXK1* expression is rather controlled by a complex mechanism including a feedback system and is, unlike *SUC2*, not regulated by catabolite repression.

## Discussion

In this study, we demonstrate that the Mig1 localization dynamics exhibit a short- and a long-term response after addition of hexoses to ethanol-grown cells. We also show that the short-term Mig1 response can be facilitated through any hexose phosphorylating activity independent of the nature of hexokinases, whereas long-term nucleocytoplasmic shuttling of Mig1 specifically requires Hxk2 and/or Hxk1 depending on the available hexose. The initial Mig1 response is associated with strict repression of the *SUC2* and *HXK1* promoters, whereas the long-term response is characterized by pulsatile behavior of Mig1 associated with partial derepression from the *SUC2* and *HXK1* promoters.

The two phases in the Mig1 localization response to hexose addition are distinguished by their timing, i.e., short-term and long-term, by their requirement for hexose phosphorylating enzymes as well as Mig1 behavior during the phases. In the immediate, short-term phase, the strength of Mig1 nuclear localization appears to be proportional to the hexose phosphorylating activity of the cell. The short-term response of Mig1 was more pronounced in the presence of fructose compared to glucose, which reflects the preference of Hxk1 and Hxk2 to phosphorylate fructose in vivo (Lobo and Maitra 1977). It appears that any of the three hexose phosphorylating enzymes can support the short-term response in response to glucose and mannose, while Glk1 cannot support a response to fructose. This is expected since Glk1 cannot phosphorylate fructose.

The long-term response is characterized by nucleocytoplasmic shuttling of Mig1 which specifically requires the presence of Hxk2 and/or Hxk1. The nucleocytoplasmic shuttling of Mig1 observed by us and others (Dalal et al. 2014; Lin et al. 2015), is a different phenomenon than the observation of mobile and immobile populations of Mig1 monomers and multimers moving across the nuclear envelope observed by Wollmann et al. through single-molecule microscopy (Wollman et al. 2017). Although both processes may be related, nucleocytoplasmic shuttling as observed in this work involves the majority of the cellular Mig1 protein and happens on the timescale of minutes, whereas the movement of Mig1 clusters across the nuclear envelope involves only a few molecules and happens at sub-second timescales. It is unlikely that the balance between the mobile and immobile fractions causes pulsatile nucleocytoplasmic shuttling observed at the cellular level.

Although biphasic behavior of the catabolite repression response has been reported previously (De Winde et al. 1996; Sanz et al. 1996), we could demonstrate for the first time that Mig1 localization also exhibits biphasic behavior and that nucleocytoplasmic shuttling of Mig1 is associated with the later phase of the response. It seems that the mechanism governing the short-term response of Mig1 is different from the one required for nucleocytoplasmic shuttling since the short-term response of Mig1 could be facilitated by the kinase activity of Glk1 in the absence of the hexokinases (Figs. 2a, 3a). In contrast, nucleocytoplasmic shuttling of Mig1 required either Hxk2 and/or Hxk1, which implies a dual function in sugar phosphorylation and gene expression control for Hxk1 as well as for Hxk2. Dual functions of sugar phosphorylating enzymes in metabolism and gene regulation are not limited to Hxk1 and Hxk2, as it has been reported for other enzymes with hexokinase activity. Gal1, which phosphorylates galactose as the first step of the galactose metabolism, has also been shown to function as a transcription regulator (Zenke et al. 1996). Several mechanisms through which the hexokinases may affect Mig1 nuclear localization have been proposed. Hxk2 may act upstream of Snf1 (Sanz et al. 2000), or shield Ser211 of Mig1 from phosphorylation by Snf1 (Kayikci and Nielsen 2015). Additionally, it has been proposed that Mig1 is required for nuclear localization of Hxk2 (Ahuatzi et al. 2004) though these observations have not been confirmed by others. More recent reports indicate a direct interaction of Mig1 with Hxk2 and possibly also Hxk1 (Vega et al. 2016).

Interestingly, the nuclear localization of Mig1 is not always correlated with carbon catabolite repression in the hexokinase mutants. Comparison of the Mig1 localization profiles and expression from the *SUC2* and *HXK1* promoter confirms that Mig1 nuclear localization is correlated with carbon catabolite repression in glucose, as reported previously (Treitel et al. 1998; DeVit and Johnston 1999) (Suppl. Fig. S9). In contrast, in mannose Mig1 is localized to the nucleus, but expression from the *SUC2* and *HXK1* promoters can be observed in the *hxk1*∆ strain (Figs. 2b, 4, Suppl. Fig. S9). Also, the average long-term nuclear localization of Mig1 was not reduced in the *hxk2*∆ strain in fructose, whereas expression from the *SUC2* and *HXK1* promoters could be observed. In contrast, Mig1 nuclear localization was reduced in the absence of Hxk1, while repression on the *SUC2* and *HXK1* promoters remained unchanged (Figs. 2b, 4, Suppl. Fig. S9). It seems that Mig1 nuclear localization is a prerequisite for catabolite repression, but that depending on the carbon source Hxk2 and/or Hxk1 are additionally required.

Adding further to the complexity of glucose repression regulation, we observed that in the *hxk2*∆ strain the *SUC2* promoter activity reaches a maximum at around 300 min after the media shift and subsequently declines for all tested hexose sugars. This points towards a feedback mechanism, in which expression of *HXK1* after the media shift leads to accumulation of Hxk1 protein which compensates for the loss of Hxk2 in repression of *SUC2* (Suppl. Fig. S9). In contrast, *HXK1* promoter activity reaches a plateau 300 min after the media shift and may not be affected by this feedback mechanism. It has been reported that *HXK1* expression is repressed exclusively by Mig1, whereas *SUC2* exhibits partially redundant repression through Mig1 and its homologue Mig2 (Lutfiyya et al. 1998). This raises the possibility that the Hxk1 protein may repress *SUC2* together with Mig2 in the absence of Hxk2.

Unrelated to glucose repression, we observed that overexpression of Hxk1 or Hxk2 in cells exposed to mannose lead to a high fraction of cell death. Our results are reminiscent of the growth defect of *tps1∆* strains where an imbalance in upper glycolysis during the transition to growth on fermentable carbon sources is observed (Van Aelst et al. 1993). Indeed, overexpression of Hxk2 causes deregulation of glycolysis during the initiation of fermentation on fructose and glucose, and produces a metabolite pattern similar to the one in *tps1∆* strains (Ernandes et al. 1998) but does not lead to a growth defect. In line with that finding, deletion of *HXK2* in a *tps1∆* background repairs the growth defect (De Winde et al. 1996). Maybe the Tps1-dependent control system is unable to rebalance glycolysis in the case of mannose, when Hxk1 or Hxk2 are overexpressed.

Further work is required to understand the sub-cellular localization of Hxk1 and Hxk2 during the short and long-term response of Mig1 to hexose availability. It remains unclear if the previously reported nuclear localization of Hxk2 (and Hxk1) coincides or co-localizes with Mig1 nuclear localization and exhibits pulsatile nucleocytoplasmic shuttling. More work is needed to understand the mechanism how catabolite repression on the *SUC2* and *HXK1* promoters can be maintained during nucleocytoplasmic shuttling of Mig1.

It has previously been shown that addition of hexoses to cells grown on non-fermentable carbons sources leads to distinguishable short- and long-term regulatory effects (De Winde et al. 1996). Here, we show that the transcription response to a change in carbon-source availability displays different dynamics for the initial short-term response than for the long-term sustained response. The occurrence of fundamental differences between short and long-term response is not novel. Previous work has shown that the initial response of the nutrient-sensing PKA pathway does not require cAMP while for the sustained response of the PKA pathway cAMP is required (Griffioen et al. 1996). This shows that it is not an uncommon characteristic in transcriptional regulation pathways that the initiation of response displays different dynamics and mechanisms from the sustained response.

The nucleocytoplasmic shuttling of Mig1 creates a situation where the active Mig1 is not tethered to the nucleus, but continuously moves between the area where the signal is generated and its destination. This feature could equip the system with higher accuracy and speed of information transfer from the cytoplasm to the nucleus. It remains an open question how the nucleocytoplasmic shuttling is regulated by external stimuli, how the hexokinases mechanistically influence the Mig1 behavior, and what the effect of increased nucleocytoplasmic shuttling is on signal transduction pathways.

Overall, the importance of biphasic behavior and nucleocytoplasmic shuttling of transcription factors in cellular processes cannot be underestimated. Studying metabolic regulation systems through single-cell observation of changes in nutrient availability has the potential to illustrate the highly dynamic behavior of transcription factors. These findings may also have implications for the understanding of other fundamental biological mechanisms.

## Electronic supplementary material

Below is the link to the electronic supplementary material.

Supplementary Information 1 (DOCX 55 kb)

Supplementary Figure 1 (EPS 38789 kb)

Supplementary Figure 2 (EPS 367 kb)

Supplementary Figure 3 (EPS 38479 kb)

Supplementary Figure 4 (TIFF 8355 kb)

Supplementary Figure 5 (EPS 2756 kb)

Supplementary Figure 6(EPS 436 kb)

Supplementary Figure 7 (EPS 26723 kb)

Supplementary Figure 8 (EPS 31105 kb)

Supplementary Figure 9 (EPS 2357 kb)

Supplementary Movie 1 (AVI 2123 kb)

Supplementary Movie 2 (AVI 10137 kb)

Supplementary Movie 3 (AVI 7266 kb)
