# Supplementary material for: Mig1 localization exhibits biphasic behavior which is controlled by both metabolic and regulatory roles of the sugar kinases
Source: Mol Genet Genomics. 2020 Sep 19;295(6):1489–500. doi: 10.1007/s00438-020-01715-4 (PMC7524853; doi:10.1007/s00438-020-01715-4)
Supplement: Supplementary file 1 — Supplementary Information 1 (DOCX 55 kb) [file 438_2020_1715_MOESM1_ESM.docx]

# Supplementary information for:

Mig1 localization exhibits biphasic behavior which is controlled by both metabolic and regulatory roles of the sugar kinases.

Gregor W. Schmidt1,*, Niek Welkenhuysen2,3,*, Tian Ye2, Marija Cvijovic^3^, and Stefan Hohmann2,4,§

*equal contribution

^§^corresponding author, [Stefan.hohmann@chalmers.se](mailto:Stefan.hohmann@chalmers.se)

1Department of Biosystems Science and Engineering, ETH Zurich, Basel, Switzerland

2Department of Chemistry and Molecular Biology, University of

Gothenburg, Sweden

3Department of Mathematical Sciences, University of Gothenburg and Chalmers University of Technology, Sweden

4Department of Biology and Biological Engineering, Chalmers University

of Technology, Sweden

# Supplementary Experimental information

## Design of fluorescent *SUC2*- and *HXK1*-promoter reporter constructs

The constructs are based on the integrative plasmid FRP1432 (Gnuegge, Liphardt, and Rudolf 2016) which contains the fluorescent protein Citrine destabilized with an Adh1-tail under the control of the *TDH3* promoter and the *CYC1* terminator. The plasmid was first modified by replacing the *CYC1* terminator with the *ACT1* terminator. The *ACT* terminator was amplified from genomic DNA using FRO3109 and FRO3110. The PCR product was digested using XhoI/KpnI and cloned into FRP1432 digested with XhoI/KpnI. Correct integration was checked by sequencing.

The *HXK1* promoter was amplified by PCR using primers FRO3984 and FRO3985 on W303 genomic DNA amplifying the region -984 bp to 0 bp relative to the start codon of the *HXK1* ORF. The PCR product was digested using SpeI/XbaI and cloned into the modified FRP1432 vector digested with SpeI/XbaI. Correct integration was checked by sequencing. The construct expressing Citrine under the control of the *SUC2* promoter was constructed by performing PCR using primers FRO3986 and FRO3987 on W303 genomic DNA amplifying the region -700 bp to 0 bp relative to the start codon of the *SUC2* ORF. The PCR product was digested using SpeI/XbaI and cloned into the modified FRP1432 vector digested with SpeI/XbaI. Correct integration was checked by sequencing.

Plasmids were linearized using AscI and transformed into W303 wild type, *hxk1*∆, *hxk2*∆ and *hxk1*∆*hxk2*∆ strains using the lithium-acetate method (Gietz and Woods 2002) to integrate the constructs into the genome. Correct integration into the genome at the *URA3* site was tested by colony PCR using primers FRO2167, FRO2168, FRO2169 and FRO2170 as described (Gnuegge, Liphardt, and Rudolf 2016). All primer sequences can be found in Supplementary table 3.

## Design of hexokinase/glucokinase overexpression plasmids

All sugar kinase overexpression plasmids are based on the p414GPD vector (Mumberg, Müller, and Funk 1995), which contains a multiple cloning site (MCS) flanked by the *TDH3* promoter (also called *GPD* promoter) and the *CYC1* terminator. The open reading frames of *HXK1*, *HXK2* and *GLK1* were obtained from yeast genomic DNA by PCR and cloned into the MCS of p414GPD to obtain plasmids FRP2095, FRP2096 and FRP2097, respectively. To be able to approximate the expression level of *HXK1, HXK2* and *GLK1* from the plasmid in single cells, we inserted a second expression cassette containing the violet-excitable yellow-fluorescent protein mAmetrine destabilized by an Adh1-tail. The mAmetrine ORF was also flanked by a *TDH3* promoter and a *CYC1* terminator. The mAmetrine expression cassette was obtained from the previously published plasmid FRP1479 (Gnuegge, Liphardt, and Rudolf 2016), and cloned into p414GPD, FRP2095, FRP2096 and FRP2097 using the EagI sites to yield plasmids FRP2137, FRP2138, FRP2139 and FRP2140, respectively. We selected only clones where the expression cassettes of the sugar kinases and mAmetrine were arranged such that the ORFs point into the same direction. Correct assembly of the plasmids was confirmed by sequencing.

## Fabrication of microfluidic device for imaging experiments

The microfluidic chip (adapted from (Frey et al. 2015) was designed in AutoCAD (Autodesk, Mu¨nchen, Germany). The chip consists of one layer of poly (dimethylsiloxane) (PDMS, Sylgard 184, Dow Corning Corp., USA), attached to a cover glass (thickness: 150 *µ*m, size: 24 mm x 60 mm). The PDMS layer was casted from its respective mold that was fabricated by using well established photolithographic and dry etching processes on 4-inch silicon wafers. The first layer was fabricated by patterning of a dry etching mask using ma-P 1240 photoresist (micro resist technology GmbH, Berlin-Köpenick, Germany) according to the manufacturer’s instructions. Dry etching was performed on an Ionfab 300 (Oxford instruments, Abingdon, United Kingdom). The thickness of the first layer defines the gap between the glass and the clamping pad, and was kept at 3.75 *µ*m for *S. cerevisiae*. Three layers of SU-8 were patterned on top of the dry etched silicon substrate to complete the microfluidic design. We followed the manufacturers datasheet (Microchem Corp.,Westborough, USA) to structure each of the SU-8 layers. In brief, SU-8 was spin-coated at the desired thickness and soft-baked on a hotplate. The SU-8 was then exposed through a transparency mask (Selba S.A., Versoix, Switzerland) using a UV mask aligner (MA/BA8-Gen3 mask aligner, SUSS MicroTec AG, Garching, Germany) and baked on a hotplate for cross-linking. Unexposed SU-8 was removed in the SU-8 developer. The molds were coated with trichloro(1H,1H,2H,2H-perfluoro-octyl)silane (Sigma-Aldrich, Switzerland) in a vapor silanization process. 25g of freshly mixed (1:10 ratio) and degassed PDMS was poured onto the mold. After curing in a convection oven at 80*◦*C for at least 2 hours, the PDMS was removed from the silicon wafer. Single chips were cut from the PDMS sheet and access holes were punched at inlet and outlet sites.

## Setup of microfluidic imaging experiments

The cells were inoculated in 5 mL of CSM media (lacking the appropriate amino acids for auxotrophic selection) supplemented with 3% ethanol and 0.05 % glucose from freshly streaked (2-4 days old) plates and incubated in a shaker at 270 rpm and 30*◦*C for at least 8 hours on the day before the experiment. In the evening cells were diluted to OD600 0.5 or 1 in fresh media for overnight incubation. On the next morning, the cell concentration was measured using a Z2 Coulter Counter (Beckman Coulter, Nyon, Switzerland). Typical cell concentrations ranged from 5 to 15*106 cells/ml. 1 ml of culture was transferred to a 1.5 ml tube and spun at 1000 g for 2 min and the appropriate amount of supernatant was removed to yield a final cell concentration of 20*106 cells/ml. The cells were suspended in the remaining media using a Vortex Genie 2 (Scientific industries, New York, USA) for 10 sec at speed setting 4. The microfluidic PDMS device and a glass slide (thickness 150-*µ*m, 24 mm x 60 mm) were rinsed with acetone, isopropanol, deionized water and dried using a nitrogen gun. Both were then placed in a sterile cell culture hood for chip loading. 0.4 *µ*l of cell solution was transferred onto each culturing area by using a conventional pipette. The cover glass was placed on top and slightly pressed down onto the PDMS. Due to the adhesion forces between PDMS and glass, a reversible bond was formed. The device was then transferred to the microscope. First, the vacuum channel was attached to the in-house vacuum supply to facilitate adhesion of the PDMS chip to the glass slide. Next, the outlet tubing and the first inlet tubing of the syringe filled with the ethanol-containing media was attached and the flow through the device (10 *µ*l/min) was started. We waited for the device to fill with media and for ethanol-containing media to emerge from the second inlet. Only then we did attach the second inlet tubing of the syringe filled with the hexose containing media. We raised the flow of the ethanol syringe to 50 *µ*l/min and applied a negative flow of -10 *µ*l/min for 10 min on the hexose syringe to prime the tubing of the hexose syringe with ethanol-containing media and prevent leakage of hexose containing media into the chip. After that, the flow on the ethanol and hexose syringes was set to 10 *µ*l/min and 0 *µ*l/min, respectively. Bubbles introduced during loading were removed in less than 1 h. The microfluidic device was placed in a custom-made chip holder and fixed using nail polish (Maybelline, L’Oreal Suisse S.A, Vernier, Switzerland). A detailed protocol on how to set up this microfluidic device and how it can be re-used several times has been published recently (Schmidt, Frey, and Rudolf 2018). Microfluidic syringe pumps (Cetoni GmbH, Korbussen, Germany) were used for continuous perfusion and media switching. The microfluidic pumps were controlled through YouScope (Lang, Rudolf and Stelling 2012). The flow profile for the syringe pumps during media shift experiments in the microfluidic long-term culture of yeast was programmed as follows:

| **Time**  **[min]** | **Flow rate ethanol syringe**  **[*µ*l/min]** | **Flow rate hexose syringe**  **[*µ*l/min]** |
| --- | --- | --- |
| -240 | 10 | 0 |
| 0 | 0 | 50 |
| 10 | 0 | 10 |
| 480 | 50 | -10 |
| 490 | 10 | 0 |

The time is given relative to the time point of media shift as throughout the the paper.

## Spotting assay

Cells were inoculated in 5 mL of CSM media (lacking amino acids for auxotrophic selection) supplemented with 2% ethanol and 0.05 % glucose from freshly streaked (2-4 days old) plates and incubated in a shaker at 270 rpm and 30°C for at least 8 h on the day before the experiment. In the evening cells were diluted to OD_600_ 0.5 in 5 mL of fresh media for overnight incubation. On the morning of the next day, the OD_600_ typically ranged from 2.6 to 5.8. The OD_600_ was adjusted to 1 for each strain by adding CSM media (lacking amino acids for auxotrophic selection) supplemented with only 2% ethanol. A 3-fold serial dilution of the OD adjusted cultures was prepared in a 96-well plate using CSM media (lacking amino acids for auxotrophic selection) supplemented with only 2% ethanol as the diluent. 5µl of diluted culture were spotted onto agar plates with CSM media supplemented with 2% hexose. The plates were incubated at 30°C for two days before pictures were taken.

# Supplementary tables

## Supplementary table 1: Yeast strains used in this study

| Name | Description | Genotype | Source |
| --- | --- | --- | --- |
| YSH202 | W303-1A | *MATa leu2-3/112 ura3-1 trp1-1 his3-11/15 ade2-1 can1-100 GAL SUC2* | (Thomas and Rothstein 1989) |
| YSH297 | *hxk1∆* | YSH202, *hxk1Δ::HIS3* | (Hohmann et al. 1993) |
| YSH310 | *hxk2∆* | YSH202, *hxk2Δ::LEU2* | (Hohmann et al. 1993) |
| YSH327 | *hxk1∆hxk2∆* | YSH202, *hxk1Δ::HIS3 hxk2Δ::LEU2* | (Hohmann et al. 1993) |
| FRY2166 | Nrd1-mCherry, Mig1-GFP | YSH202, *NRD1-mCherry::Hyg MIG1-GFP::KanMX* | This study |
| FRY2167 | *hxk1∆,* Nrd1-mCherry, Mig1-GFP | YSH297, *NRD1-mCherry::Hyg MIG1-GFP::KanMX* | This study |
| FRY2168 | *hxk2∆,* Nrd1-mCherry, Mig1-GFP | YSH310, *NRD1-mCherry::Hyg MIG1-GFP::KanMX* | This study |
| FRY2169 | *hxk1∆hxk2∆,* Nrd1-mCherry, Mig1-GFP | YSH327, *NRD1-mCherry::Hyg MIG1-GFP::KanMX* | This study |
| FRY2279 | *hxk1∆hxk2∆,* Nrd1-mCherry, Mig1-GFP, *TDH3*p-MCS-*CYC1*t, *TDH3*p-mAmetrine-CYC1t | FRY2169 transformed with pFRP2137 | This study |
| FRY2280 | *hxk1∆hxk2∆,* Nrd1-mCherry, Mig1-GFP, *TDH3*p-*HXK1*-*CYC1*t, *TDH3*p-mAmetrine-*CYC1*t | FRY2169 transformed with pFRP2138 | This study |
| FRY2281 | *hxk1∆hxk2∆,* Nrd1-mCherry, Mig1-GFP, *TDH3*p-*HXK2*-*CYC1*t, *TDH3*p-mAmetrine-*CYC1*t | FRY2169 transformed with pFRP2139 | This study |
| FRY2282 | *hxk1∆hxk2∆,* Nrd1-mCherry, Mig1-GFP, *TDH3*p-*GLK1*-*CYC1*t, *TDH3*p-mAmetrine-*CYC1*t | FRY2169 transformed with FRP2140 | This study |
| FRY2325 | *HXK1*p-Citrine-*ACT1*t | YSH202, *HXK1*p-Citrine(A206K) BamHIlin ker\Adh1tail-*ACT1*t::*URA3* | This study |
| FRY2328 | *hxk1∆,* *HXK1*p-Citrine-*ACT1*t | YSH297, *HXK1*p-Citrine(A206K) BamHIlin ker\Adh1tail-*ACT1*t::*URA3* | This study |
| FRY2331 | *hxk2∆,* *HXK1*p-Citrine-*ACT1*t | YSH310, *HXK1*p-Citrine(A206K) BamHIlin ker\Adh1tail-*ACT1*t::*URA3* | This study |
| FRY2334 | *hxk1∆hxk2∆,* HXK1p-Citrine-ACT1t | YSH327, HXK1p-Citrine(A206K) BamHIlin ker\Adh1tail-ACT1t::URA3 | This study |
| FRY2326 | *SUC2*p-Citrine-*ACT1*t | YSH202, *SUC2*p-Citrine(A206K) BamHIlin ker\Adh1tail-*ACT1*t::*URA3* | This study |
| FRY2329 | *hxk1∆,* *SUC2*p-Citrine-*ACT1*t | YSH297, *SUC2*p-Citrine(A206K) BamHIlin ker\Adh1tail-*ACT1*t::*URA3* | This study |
| FRY2332 | *hxk2∆,* *SUC2*p-Citrine-*ACT1*t | YSH310, *SUC2*p-Citrine(A206K) BamHIlin ker\Adh1tail-*ACT1*t::*URA3* | This study |
| FRY2335 | *hxk1∆hxk2∆,* *SUC2*p-Citrine-*ACT1*t | YSH327, *SUC2*p-Citrine(A206K) BamHIlin ker\Adh1tail-*ACT1*t::*URA3* | This study |

## Supplementary table 2: Plasmids used in this study

| Name | Backbone | insert | Selection Marker | Source |
| --- | --- | --- | --- | --- |
| p414GPD | p414GPD | *TDH3*p-MCS-*CYC1*t | *TRP1* | (Mumberg, Müller, and Funk 1995) |
| pFRP2095 | p414GPD | *TDH3*p*-HXK1-CYC1*t | *TRP1* | this study |
| pFRP2096 | p414GPD | *TDH3*p-*HXK2*-*CYC1*t | *TRP1* | this study |
| pFRP2097 | p414GPD | *TDH3*p-*GLK1*-*CYC1*t | *TRP1* | this study |
| pFRP2135 | pRG206 | *HXK1*p-Citrine(A206K) BamHIlinker *ADH1*tail-*ACT1*t | *URA* | Backbone: (Gnuegge, Liphardt, and Rudolf 2016) |
| pFRP2136 | pRG206 | *SUC2*p-Citrine(A206K) BamHIlinker *ADH1*tail-*ACT1*t | *URA* | Backbone: (Gnuegge, Liphardt, and Rudolf 2016) |
| pFRP2137 | p414GPD | *TDH3*p-MCS-*CYC1*t, *TDH3*p-mAmetrine BamHIlinker *ADH1*tail-*CYC1*t | *TRP1* | this study |
| pFRP2138 | p414GPD | *TDH3*p-*HXK1*-*CYC1*t, *TDH3*p-mAmetrine BamHIlinker *ADH1*tail-*CYC1*t | *TRP1* | this study |
| pFRP2139 | p414GPD | *TDH3*p-*HXK2*-*CYC1*t, *TDH3*p-mAmetrine BamHIlinker *ADH1*tail-*CYC1*t | *TRP1* | this study |
| pFRP2140 | p414GPD | *TDH3*p-*GLK1*-*CYC1*t, *TDH3*p-mAmetrine BamHIlinker *ADH1*tail-*CYC1*t | *TRP1* | this study |

## Supplementary table 3: Primers used in this study

| Name | Target | Purpose | Direction | Sequence |
| --- | --- | --- | --- | --- |
| HXK1/BamHI/FW | HXK1 ORF | amplification from gDNA for cloning | forward | ATCTACGGATCCGACCG |
| HXK1/EcoRI/RV | HXK1 ORF | amplification from gDNA for cloning | reverse | CTAGGGAATTCTTAAG |
| HXK2/BamHI/FW | HXK2 ORF | amplification from gDNA for cloning | forward | ATCTACGGATCCATCGA |
| HXK2/EcoRI/RV | HXK2 ORF | amplification from gDNA for cloning | reverse | CTAGGGAATTCGGTAT |
| GLK1/BamHI/FW | GLK1 ORF | amplification from gDNA for cloning | forward | ATCTACGGATCCATGTC |
| GLK1/EcoRI/  RV | GLK1 ORF | amplification from gDNA for cloning | reverse | CTAGGGAATTCTCATG |
| FRO3984 | HXK1 promoter | amplification from gDNA for cloning | forward | GATTATCTAGACCATGG |
| FRO3985 | HXK1 promoter | amplification from gDNA for cloning | reverse | GGATCAACTAGTCTTAT |
| FRO3986 | SUC2 promoter | amplification from gDNA for cloning | forward | TATGATTCTAGAACATA |
| FRO3987 | SUC2 promoter | amplification from gDNA for cloning | reverse | TATTATACTAGTCATAT |
| FRO2167 | upstream of pRGXXX integration site HR1 | test genomic integration | forward | AGCTTTGATGTTGTG |
| FRO2168 | downstream of pRGXXX integration site HR1 | test genomic integration | reverse | CATATTTGAGAAGAT |
| FRO2169 | upstream of pRGXXX integration site HR2 | test genomic integration | forward | CTGTGTGAAATTGTT |
| FRO2170 | downstream of pRGXXX integration site HR2 | test genomic integration | reverse | ACGGTTCATCATCTC |
| FRO3109 | ACT1 terminator | amplification from gDNA for cloning | forward | GATCCTCGAGTCTCTG |
| FRO3110 | ACT1 terminator | amplification from gDNA for cloning | reverse | TAGCGGTACCTACACG |

## Supplementary table 4: Excitation filter / dichroic / emission filter combinations used for long-term imaging of fluorescent protein fusions as described in materials and methods. The light power setting of the epifluorescence excitation light source was adjusted such that the light intensity was 2.91 W cm−2 for all imaging channels.

| Fluorescent protein | Excitation filter | Beam splitter | Emission filter | SpectraX LED | SpectraX power | Exposure time |
| --- | --- | --- | --- | --- | --- | --- |
| mAmetrine | 438/24 nm | 495LPXR | 525/50 nm | blue | 8% | 400 ms |
| GFP | 488/6 nm | 495LPXR | 525/50 nm | cyan | 25% | 200 ms |
| Citrine | 504/12 nm | HC-BS520 | 542/22 nm | green | 100% | 500 ms |
| mCherry | 561/4 nm | HC-BS573 | 605/40 nm | green | 42% | 300 ms |

# Supplementary movies

## Supplementary movie 1

## ”SupplMovie1.avi”

Time-lapse movie of wild type cells during shift from 510 mM ethanol to 220 mM  mannose (0 min) back to 510 mM ethanol (720 min). Images from left to right: Out-of-focus brightfield channel; Mig1-GFP channel; Nrd1-mCherry channel; Overlay of all three channels. The fluorescence images of Mig1-GFP and Nrd1-mCherry were contrast adjusted through linear re-scaling between intensity values of 200 and 700 AU.

## Supplementary movie 2

## ”SupplMovie2.avi”

 Time-lapse movie of *hxk1∆hxk2∆* cells overexpressing Hxk1 and the blue excitable yellow-fluorescent protein mAmetrine from similar expression cassettes with *TDH3*-promoters and *CYC1*-terminators (plasmid FRP2138), during shift from 510 mM ethanol to 220 mM mannose (0 min) back to 510 mM ethanol (720 min). Images from left to right: Out-of-focus brightfield channel; Mig1-GFP channel; Nrd1-mCherry channel; Overlay of previous three channels; mAmetrine channel. The fluorescence images of Mig1-GFP and Nrd1-mCherry were contrast-adjusted through linear re-scaling between intensity values of 200 and 700 AU. The fluorescence images of mAmetrine were contrast-adjusted through linear re-scaling between intensity values of 200 and 1000 AU.

## Supplementary movie 3

## ”SupplMovie3.avi”

 Time-lapse movie of *hxk1∆hxk2∆* cells overexpressing Hxk2 and the blue excitable yellow-fluorescent protein mAmetrine from similar expression cassettes with *TDH3*-promoters and *CYC1*-terminators (plasmid FRP2139), during shift from 510 mM ethanol to 220 mM mannose (0 min) back to 510 mM ethanol (720 min). Images from left to right: Out-of-focus brightfield channel; Mig1-GFP channel; Nrd1-mCherry channel; Overlay of previous three channels; mAmetrine channel. The fluorescence images of Mig1-GFP and Nrd1-mCherry were contrast-adjusted through linear re-scaling between intensity values of 200 and 700 AU. The fluorescence images of mAmetrine were contrast-adjusted through linear re-scaling between intensity values of 200 and 1000 AU.

# Supplementary figures

## Supplementary figure 1 “Fig_S1.eps”

##

Mig1-GFP localization during shift from 510 mM ethanol to 220 mM glucose, fructose or mannose (0 min) back to 510 mM ethanol (720 min) for wild type (WT) (a-c), *hxk1∆* (d-f), *hxk2∆* (g-i) and *hxk1∆hxk2∆* (j-l) strains. Single cell traces of Mig1 localization over time are shown in gray. 7 random tracks are marked with colors. The average of all cells is shown in black. Media switches are indicated by vertical black lines.

## Supplementary figure 2 “Fig_S2.eps”

##

Glucokinase activity in wild type (WT) and in a *hxk1∆hxk2∆* double mutant transformed with either empty plasmid or plasmids expressing *HXK1*, *HXK2* or *GLK1* under control of the *TDH3*-promoter and *CYC1*-terminator grown in 4% glucose YNB(-Trp). Bars represent mean of three replicate measurements. Single measurements are represented by dots.

## Supplementary figure 3 “Fig_S3.eps”

##

Mig1-GFP localization during shift from 510 mM ethanol to 220 mM glucose, mannose or fructose (0 min) back to 510 mM ethanol (720 min) in a *hxk1∆hxk2∆* double mutant transformed with either empty plasmid or plasmids expressing *HXK2*, *HXK1* or *GLK1* under control of the *TDH3*-promoter and *CYC1*-terminator. Single cell traces of Mig1-GFP localization over time are shown in gray. 7 random tracks are marked with colors. The average of all cells is shown in black. Media switches are indicated by vertical black lines.

## Supplementary figure 4 “Fig_S4.tif”

Viability and growth of an *hxk1∆hxk2∆* strain overexpressing *HXK1*, *HXK2* or *GLK1* after shift from ethanol to either 220 mM glucose (a), mannose (b) or fructose (c). The *hxk1∆hxk2∆* strain was transformed with either empty plasmid or plasmids expressing *HXK1*, *HXK2* or *GLK1* under control of the *TDH3*-promoter and *CYC1*-terminator. Cells were pre-cultured in YNB(-Trp) supplemented with 2% ethanol and 0,05% glucose and dilution series were spotted at the indicated OD onto YNB(-Trp) agar plates supplemented with the corresponding hexose and imaged after 2 days at 30°C.

## Supplementary figure 5 “Fig_S5.eps”

##

Correlation between Mig1-GFP localization and activity of the *TDH3*-promoter driving the expression of different sugar kinases in an *hxk1∆hxk2∆* strain. Cells were transformed with either empty plasmid or plasmids expressing *HXK1*, *HXK2* or *GLK1* under control of the *TDH3*-promoter and *CYC1*-terminator. The plasmids additionally carried another expression cassette with the blue-excitable yellow-fluorescent protein mAmetrine which was also under the control of a *TDH3*-promoter and *CYC1*-terminator. The mAmetrine fluorescent signal was used to estimate the activity of the *TDH3*-promoter in single cells and thus serves as a proxy for the sugar kinase expression level. The mean Mig1-GFP localization from 240-480 min after shift from ethanol to 220 mM glucose, mannose or fructose was plotted against the mean mAmetrine signal from 240-480 min after the shift. Each point represents the measurement of a single cell. A linear function (a*x + b) was fitted to the data and is represented by a dashed line.

## Supplementary figure 6 “Fig_S6.eps”

Invertase activity of wild type, *hxk1∆, hxk2∆* and *hxk1∆hxk2∆* double mutant strains 120 minutes after shift to YNB supplemented with the indicated carbon source. Bars represent mean of replicate measurements. Single measurements are represented by dots. Invertase activity correlates with fluorescence signal from *SUC2*p-Citrine expression (Figure 4a).

## Supplementary figure 7 “Fig_S7.eps”

##

*SUC2*-promoter activity during shift from 510 mM ethanol to 220 mM glucose, mannose or fructose (0 min) back to 510 mM ethanol (720 min) in wild type (WT) (a-c), *hxk1∆* (d-f), *hxk2∆* (g-i) and *hxk1∆hxk2∆* (j-l) strains. Single cell traces of the signal obtained from the yellow-fluorescent protein Citrine driven by a *SUC2*-promoter are shown in gray. Seven random tracks are marked with colors. The average of all cells is shown in black. Media switches are indicated by vertical black lines

## Supplementary figure 8 “Fig_S8.eps”

##

*HXK1-*promoter activity during shift from 510 mM ethanol to 220 mM glucose, mannose or fructose (0 min) back to 510 mM ethanol (720 min) in WT (a-c), *hxk1∆* (d-f), *hxk2∆* (g-i) and *hxk1∆hxk2∆* (j-l) strains. Single-cell traces of the signal obtained from the fluorescent protein Citrine under control of the *HXK1* promoter are shown in gray. Seven random tracks are marked with colors. The average of all cells is shown in black. Media switches are indicated by vertical black lines.

## Supplementary figure 9 “Fig_S9.eps”

##

Overlay of mean Mig1-GFP localization, mean *SUC2*-promoter and mean *HXK1*-promoter activity during shift from 510 mM ethanol to 220 mM glucose, mannose or fructose (0 min) back to 510 mM ethanol (720 min) in wild type (WT) (a-c), *hxk1∆* (d-f), *hxk2∆* (g-i) and *hxk1∆hxk2∆* (j-l) strains. Data were assembled from Supplementary figures 3 & 5 & 6. Media switches are indicated by vertical black lines.

# References for supplementary information

Frey, Olivier, Fabian Rudolf, Gregor W Schmidt, and Andreas Hierlemann. 2015. “Versatile, Simple-to-Use Microfluidic Cell-Culturing Chip for Long-Term, High-Resolution, Time-Lapse Imaging.” *Analytical Chemistry* 87 (8): 4144–51. https://doi.org/10.1021/ac504611t.

Gietz, R Daniel, and Robin A Woods. 2002. “Transformation of Yeast by Lithium Acetate/Single-Stranded Carrier DNA/Polyethylene Glycol Method.” *Methods in Enzymology* 350: 87–96. https://doi.org/10.1016/S0076-6879(02)50957-5.

Gnuegge, Robert, Thomas Liphardt, and Fabian Rudolf. 2016. “A Shuttle Vector Series for Precise Genetic Engineering of Saccharomyces Cerevisiae.” *Yeast* 33 (3): 83–98. https://doi.org/10.1002/yea.

Hohmann, Stefan, Maria José Neves, Wim de Koning, Rafael Alijo, José Ramos, and Johan M Thevelein. 1993. “The Growth and Signalling Defects of the Ggs1 (Fdp1/Byp1) Deletion Mutant on Glucose Are Suppressed by a Deletion of the Gene Encoding Hexokinase PII.” *Current Genetics* 23 (4): 281–89. https://doi.org/10.1007/BF00310888.

Lang, Moritz, Fabian Rudolf, and Jörg Stelling. 2012. “Use of Youscope to Implement Systematic Microscopy Protocols.” *Current Protocols in Molecular Biology* 1 (SUPPL.98): Unit 14.21.1--23. https://doi.org/10.1002/0471142727.mb1421s98.

Mumberg, Dominik, Rolf Müller, and Martin Funk. 1995. “Yeast Vectors for the Controlled Expression of Heterologous Proteins in Different Genetic Backgrounds.” *Gene* 156 (1): 119–22. https://doi.org/10.1016/0378-1119(95)00037-7.

Schmidt, Gregor W, Olivier Frey, and Fabian Rudolf. 2018. “The CellClamper: A Convenient Microfluidic Device for Time-Lapse Imaging of Yeast.” *Methods Mol. Biol.* 1672: 537–55. https://doi.org/10.1016/j.cell.2013.01.002.

Thomas, Barbara J, and Rodney Rothstein. 1989. “Elevated Recombination Rates in Transcriptionally Active DNA.” *Cell* 56 (4): 619–30. https://doi.org/10.1016/0092-8674(89)90584-9.
