# Supplementary figures and images for: Mig1 localization exhibits biphasic behavior which is controlled by both metabolic and regulatory roles of the sugar kinases
Source: Mol Genet Genomics. 2020 Sep 19;295(6):1489–500. doi: 10.1007/s00438-020-01715-4 (PMC7524853; doi:10.1007/s00438-020-01715-4)

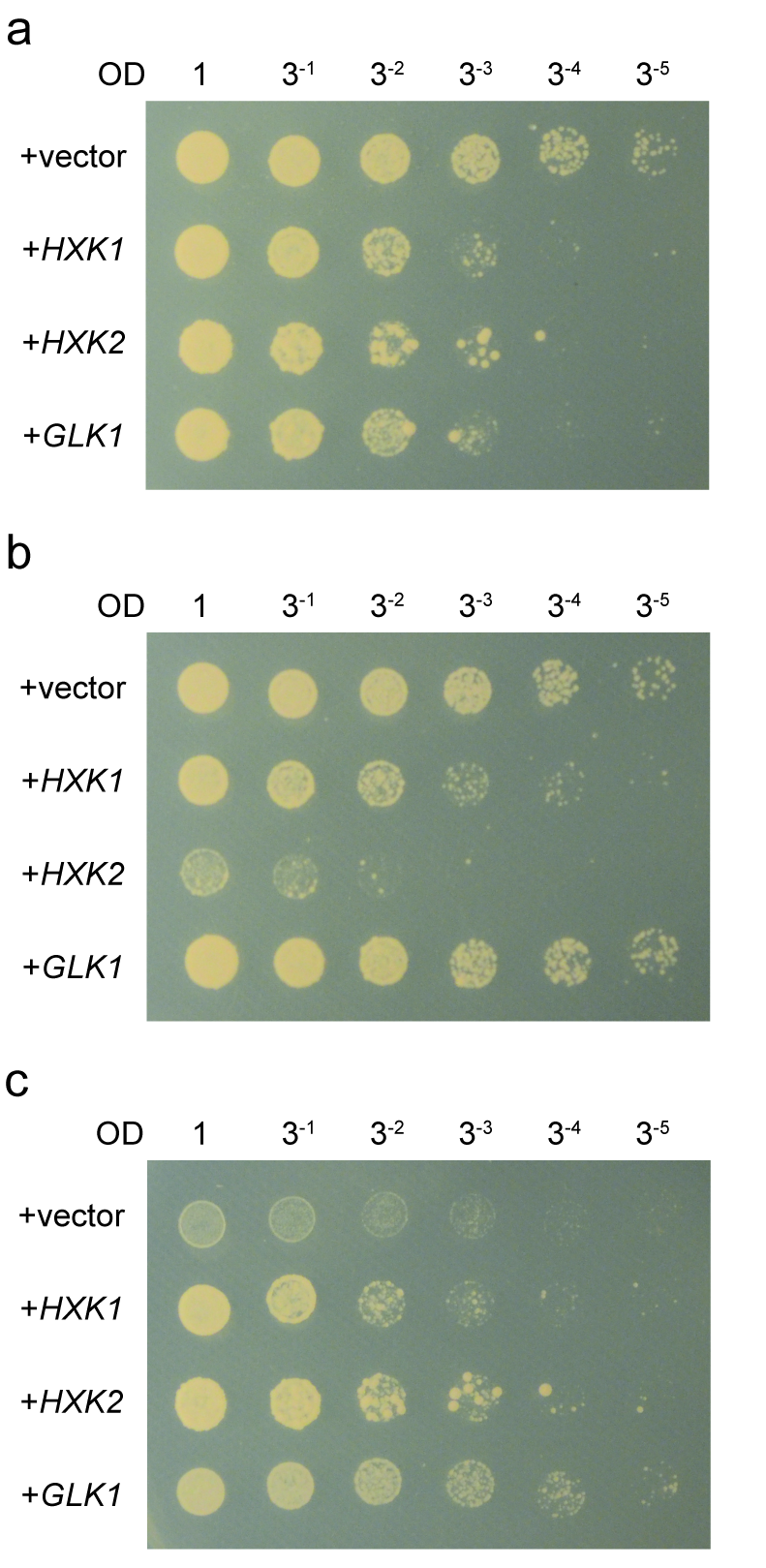

Supplement: Supplementary file 5 — Supplementary Figure 4 (TIFF 8355 kb) [file 438_2020_1715_MOESM5_ESM.tif]
